# Clinical Outcomes of Incidental Dural Tears During Lumbar Microdiscectomy

**DOI:** 10.7759/cureus.14360

**Published:** 2021-04-08

**Authors:** Uzay Erdoğan, Aykut Akpinar

**Affiliations:** 1 Neurosurgery, University of Health Sciences, Bakırköy Prof. Dr. Mazhar Osman Training and Research Hospital for Neurology, Neurosurgery and Psychiatry, Istanbul, TUR; 2 Neurosurgery, University of Health Sciences, Haseki Research and Training Hospital, Istanbul, TUR

**Keywords:** complication, dural tear, incidental, lumbar disc surgery

## Abstract

Background: A dural tear (DT) is the most commonly encountered complication during lumbar spine surgery. The incidence of DT increases depending on the complexity of the surgical procedure and the presence of a DT is related to a poor outcome and patient satisfaction.

Objectives: This study aimed to determine the incidence and clinical outcomes of DTs in those patients who undergo lumbar disc surgery.

Methods: We retrospectively reviewed consecutive patients who underwent surgery for the management of a primary single-level lumbar disc herniation at a single institution between 2004 and 2014. Among the studied population, those with DTs were included in the study group. An age- and sex-matched group of randomly selected patients who underwent the same level and type of lumbar spine surgery, but did not develop DTs, were assigned as the control group. The outcomes were compared at 12 months postoperatively between the groups.

Results: A total of 5,476 consecutive patients (2,608 female, 2,868 male; mean age, 54 ± 11.45 [range, 21-86] years) underwent surgery for primary single-level lumbar disc herniation. DT was noted in 192 (2.85%) cases. Of these, 102 patients with complete data were included in the DT group. The DT group had a significantly increased length of hospital stay (p = 0.001). Also, the duration of bed rest in the hospital was significantly higher in patients wherein DT was repaired using hemostatic material and fibrin glue, compared to the patients with primary closure with suturing of the tear.

Conclusion: Incidental DTs, if recognized and treated appropriately, will not lead to poor clinical results and do not adversely impact postoperative outcomes.

## Introduction

An incidental dural tear (DT) is the most commonly encountered complication of lumbar decompressive and reconstructive spine surgery, with a reported prevalence ranging from 3% to 16% [1,2]. The wide variance of DT incidence is multifactorial, due to the derivation of reported prevalence ratios from questionnaires and multicenter studies, the variations of patient characteristics, types of revision surgery, surgical procedures, disease progression, and the consecutive spinal surgery cases [1,3]. Anatomical variations (bony protuberances and ossification of the ligamentum flavum), adhesions and fibrosis, surgical technique, surgeon’s experience, patient age, type of surgical procedure, the intensity of the decompression, complexity of the interventions, and revision surgery are among the risk factors in the development of incidental DTs [4-6]. Furthermore, while the complexity of the spinal procedures increases, the incidence of DT rises, regardless of the experience of the operating surgeon.

Although the clinical outcomes in patients with DTs are often not reported, the outcomes in patients undergoing lumbar disc herniation surgery have been reported as unsatisfactory in patients with incidental DTs compared to those without DT [6-8].

The most frequent symptom of an early-stage DT is a severe postural headache, often accompanied by nausea and vomiting. Radicular pain related to nerve root entrapment with a severity equal to or higher than that experienced preoperatively, and neurological deficits related to neural tissue damage frequently manifest as other early-stage symptoms [9]. Meningeal pseudocyst and fistula formation, meningitis due to contamination from the external environment, cerebrospinal fluid (CSF) leakage, and delay in wound healing are among well-known long-term outcomes of dural defects [10].

Because of the insufficient information and controversies regarding the effect of DTs on long-term outcomes at various centers, we decided to conduct a retrospective review of the incidence of DTs during single-level lumbar spine surgery and assessed the impact on patient outcomes in one center from 2004 to 2014.

## Materials and methods

Approval by the local ethics board committee was obtained prior to the collection of the study data (12.01.2016/513). This study did not require informed consent from the patients as it was a retrospective study. However, the operations had been performed in a teaching hospital, and informed consent was obtained from all patients whose clinical data could be used for educational and scientific purposes.

All records of single-level lumbar spine surgery performed from 2004 to 2014 in the Division of Spine Surgery were identified. All patients underwent lumbar decompressive and reconstructive spine surgery. The indications for surgery that lead to DT were untreatable radicular pain, motor or sensible nerve root deficits, and/or cauda symptoms. Among the study population, those with DTs were designated as the study group. An age- and gender-matched group of randomly selected patients who underwent the same level and type of lumbar spine surgery with similar indications, but did not develop DT were included as the control group. The outcomes for the two groups were compared at the end of the postoperative first year. Patients with recurrent disc herniation, degenerative lumbar stenosis, spinal deformities, and traumatic injuries, and patients who underwent instrumentation, fusion, and reoperation were excluded.

Our routine surgical protocol for single-level lumbar discectomies was as follows: Under general anesthesia, the patient was placed in the prone position on the operating table. Distance control was performed using fluoroscopy. Through a midline incision approximately 25-40 mm in length, the spinal level of interest was approached under fluoroscopy guidance, and the actual distance was determined. Under microscopic examination, a partial hemilaminectomy and resection of the ligamenta flava were performed on the side of the radiculopathy using Kerrison punch forceps. The distance between the nerve root and dura was estimated under direct vision. Thereafter, the dura and involved nerve root were retracted medially, the affected disc was approached, and the herniated disc was resected. Postoperatively, patients were prescribed flat bed rest until any related symptoms had resolved, after which they were allowed to ambulate and discharged. Patients with DT were treated with one or more procedures shown in Table 1. Patients who developed DT postoperatively were restricted from ambulation for 24-48 hours.

**Table 1:** Repair procedures for the management of DT

Treatment	Equipment
Repair of the defect with primary suturing	Atraumatic 8-, 10-, and 13-mm needles with 4/0 or 5/0 prolene or silk sutures
Covering of defects	Muscle, adipose tissue, and hemostatic material or fibrin tissue adhesives
Placement of a drain in the surgical area	
Lumbar drainage	

Statistical analysis

All statistical analyses were performed with IBM SPSS Statistics for Windows Version 20.0 (IBM Corp, Armonk, NY). The discrimination of the data was evaluated using the Kolmogorov-Smirnov test. Comparisons of age and other continuous postoperative data were performed using Student’s t-test and are presented as mean±SD. Categorical variables were evaluated with Pearson’s chi-square or Fisher’s exact test and are presented as numbers and percentages. P-values of <0.05 were considered to indicate statistical significance.

## Results

A total of 5,476 consecutive patients (2,608 female and 2,868 male; mean age, 54 ± 11.45 [range, 21-86] years) underwent elective, primary, single-level lumbar surgery between 2004 and 2014. DT occurred in 192 patients with an overall incidence of 2.85%. All procedures involved single-level surgery, and none of the patients underwent a supplementary fusion procedure.

Of the 192 patients with DT, 102 patients with sufficient data have corresponded to the study group. An age- and gender-matched group of patients who underwent the same level and type of lumbar spine surgery, but did not develop DT, were included as the control group. The DT group consisted of 53 men and 49 women, with a mean age of 49.88 ± 11.97 (range, 21-71) years. The group without DT consisted of 1,488 men and 1,377 women, with a mean age of 49.75 ± 10.99 (range, 26-86) years. The mean duration of follow-up from surgery was 12 months. Table 2 shows the demographic characteristics and surgery levels of both groups. No differences existed in terms of age, sex, and surgery level between patients with and without DT (p > 0.05).

**Table 2:** Clinical and demographic characteristics of patients with and without dural tear Values are presented as mean ± SD or n (%) NS: Not significant

	Patients with dural tear (n = 102)	Patients without dural tear (n = 2865)	
Age, years			
Male	49.90 ± 12.30	48.85 ± 11.47	NS
Female	47.42 ± 11.57	48.14 ± 10.13	NS
Gender			
Male	53 (51.96%)	52 (50.98%)	NS
Female	49 (48.03%)	50 (49.01%)	NS
Surgery Level			
L2-3 (n = 7)			
Male	1 (0.98%)	0	NS
Female	3 (2.94%)	3 (2.94%)	NS
L3-4 (n = 41)			
Male	10 (9.8%)	8 (7.8%)	NS
Female	11 (10.74%)	12 (11.76%)	NS
L4-5 (n = 85)			
Male	25 (24.5%)	24 (23.52%)	NS
Female	17 (16.6%)	19 (18.6%)	NS
L5-S1 (n = 71)			
Male	17 (16.6 %)	20 (19.6%)	NS
Female	18 (17.64%)	16 (15.6%)	NS

The repair procedures performed for those who developed DT are presented in Table 2. The repair method was primary repair using sutures in 34.3%, hemostatic material, fat, and muscle tissue in 48.0%, and fibrin tissue glue in 17.7% of the patients. Of those, 44.8% of the patients in the hemostatic material, adipose, and muscle tissue group, and 44.4% of the patients in the fibrin tissue glue group experienced CSF leaks in the following weeks. Fourteen out of 67 patients with unresolved CSF leaks for seven days despite lumbar drainage underwent reoperation. Comparison of the dura closure methods revealed that a significantly fewer number of patients developed CSF leakage with the primary suturing during the postoperative follow-up period compared to the repair with hemostatic material and fibrin glue (Table 3).

**Table 3:** Dural repair methods for patients with dural tear Values are presented as n (%) *P-value was determined between the method of comparison and primary repair **P-value was determined between the method of comparison and fibrin tissue glue CSF: Cerebrospinal fluid

Repair method	CSF leak	P-value	Lumbar drainage	P-value	Reoperation	P-value
Primary repair (n = 35)	0 (0%)	NA	0 (0%)		0 (0%)	
Hemostasis material, fat, and muscle tissue (n = 49)	22 (44.8%)	<0.05*	16 (32.6%)	<0.05*	11 (22.4%)	<0.05* <0.05**
Fibrin tissue glue (n = 18)	8 (44.4%)	<0.05*	6 (38.8%)	<0.05*	3 (16.6%)	<0.05*

The duration of bed rest for those who underwent primary repair (1.72 ± 0.56 days) was significantly shorter than that for patients who underwent repair with hemostatic material (2.7 ± 1.27 days) and fibrin glue (2.5 ± 1.24 days) (p <0.001 and p = 0.003, respectively). However, there was no significant difference between bed rest duration of patients who had dural defects covered with hemostatic material, adipose, and muscle tissue, and the bed rest of patients with dural defects covered with adhesive fibrin glue (p = 0.553). While the postoperative data of the DT and non-DT groups were compared, a significantly longer in-hospital stay was observed in patients with DTs (2.32 ± 1.15 vs 1.12 ± 0.33 days, p < 0.001) (Table 4).

**Table 4:** Dural repair method and duration of bed rest Values are presented as mean ± SD

	Bed rest duration (days)	p value
Without dural tear	1.12 ± 0.33	Reference
With dural tear	2.32 ± 1.15	<0.001
Primary suturing	1.72 ± 0.56	Reference
Hemostatic material	2.70 ± 1.27	<0.001
Fibrin glue	2.50 ± 1.24	0.003

.

## Discussion

DT is an undesirable complication of lumbar spine surgery [5]. Limited information is available regarding the incidence and outcomes of incidental DT associated with lumbar microdiscectomy. In our study, the incidence of incidental DT among 5,476 patients who underwent a single-level lumbar disc herniation procedure was 2.85%, and the need for revision surgery for managing the same was 13.7%.

A large series of degenerative lumbar procedures revealed a ratio of DT development of 7.6% for primary surgery, and 15.9% for revision cases [5]. In their four-year series of 553 consecutive patients operated for degenerative lumbar spinal stenosis, traumatic lumbar vertebral fractures, spinal tumors, and lumbar spondylolisthesis, Kalevski et al. reported their incidence of DT as 12.66% [11]. In their 10-year series of 1,359 lumbar spine operations, Wolff et al. reported an incidence of 1.7% despite their relatively lower rate of DT, their operations were comprised of microdiscectomies, a minimally invasive approach to spinal fusion [12]. The variations among the study groups and our case group might be a result of the complexity of the surgical procedure, selection bias between study groups, variations of the diagnostic tools, and surgical dexterity. Our study group consisted of a large series of patients who underwent surgery within a 10-year interval and included patients who did not undergo complex surgeries comprised single-level, primary lumbar discectomies. In addition, the study group included a homogenous group of patients who completed 12 months of follow-up.

Although most studies on the DT have been retrospective, prospective data from the Swedish Spine Registry concluded that DT is a technical problem, and if dealt with appropriately at the time of surgery, did not compromise the results of discectomies one-year postoperatively [13]. This finding was in contrast with that of a retrospective case-matched study involving 41 patients with DT in which the authors found a tendency for more reoperations, longer duration of inability to work, and more back pain [10]. Saxler et al. reported that patients with an incidental DT after lumbar disc surgery had poorer outcomes after surgery. In a retrospective study, Grannum et al. reported no difference between clinical outcomes during five years of follow-up; however, the patient group consisted of patients who had undergone discectomies or decompressive procedures, as such, the group was not homogeneous [14]. Moreover, the number of patients in their study was lower than the number of patients included in our study. Because it was a heterogeneous group, the incidence of DTs did not reflect a specific incidence. Our study compared patients with or without incidental DT following spinal procedures performed for single-level lumbar disc herniation. No significant intergroup differences in incidental DT during first-time lumbar discectomies have been reported to exist, and no impact on long-term outcomes in affected patients has been noted [15]. We also did not find a difference between the patients operated for different levels of the lumbar spine, and the incidence of DT development did not change between the groups.

A review of the literature revealed the application of different methods in the management of dural injuries, varying from primary suturing, closure of the defect with muscle, adipose tissue, or tissue adhesives, continuous subarachnoidal lumbar drainage, to bed rest. Kamenova et al. reported that the DT repair technique does not affect the revision surgery rate [16]. Herren et al. found that non-repair of DT demonstrated no negative association with treatment outcome [17]. Saxler et al. showed that during long-term follow-up, worse clinical outcomes were related to dural defects and concluded that the method of dural defect closure had no effect on clinical outcomes [10]. We suggest that, in order to achieve better clinical outcomes, primary repair of the defect is essential for the prevention of CSF leaks and the requirement for additional surgery. The gold standard for the management of a DT is primary suture repair, and our study results also supported this finding [6,7]. Although there are article reports presenting suturing as the best approach to manage DT, there are also controversial data advocating the use of glue and collagen. Furthermore, for minimally invasive procedures, in particular, suturing is a difficult-to-perform management method, hence alternative managing techniques with the easier application process are suggested. Also, in the presence of anterior and lateral tears, suturing would be impractical since approximation of tissues is difficult.

Subarachnoidal lumbar drainage is also efficient while managing dural leaks refractory to treatment. Bed rest for patients with DTs is controversial; however, we advise bed rest for postoperative 24-36 h. Traditionally, the postoperative management of these patients consisted of flatbed rest until the symptoms of posture-related headache have subsided [6]. Radcliff et al. recently reported an increased rate of medical complications in patients on bed rest for >24 h compared to patients who were confined to the bed for less than 24 h [18]. Hodges et al., in a retrospective study involving 20 patients, found that bed rest was not essential provided that the dural defect was repaired appropriately during surgery; specifically, they found that 75% of patients did not need bed rest [19]. Another study reported that the repair of DTs decreased the length of hospital stay and health care costs [2]. However, some studies have advised bed rest for a duration ranging from 2.68 to 5 days for similar patient groups [6,7,20]. A multinational analysis did not detect any difference between patients who did and did not experience DTs following lumbar discectomies [21]. In our study, the average length of hospital stay among the patients who developed DTs was 2.32 days, and the duration of hospitalization was significantly higher in this group compared to those without DT. Furthermore, the hospitalization was significantly different between the management groups, and the patients whose defects had been closed using hemostatic material and fibrin glue had a longer hospitalization compared to those who underwent closure with primary suturing. We did not experience any mortalities and morbidities, including deep venous thrombosis, embolism, hemorrhagia, or the formation of meningocele and infection.

DT is a complication that needs to be diagnosed and treated on time, that a cerebellar hemorrhagia might develop if the CSF pressure decreased gradually, and the probability of a DT had been dismissed. Establishing proper and accurate diagnostic tools, and alertness of the surgeon about the condition is of utmost importance. Testing of any smallest amount of suspected drainage for β-2-transferrin electrophoresis might provide an accurate and rapid diagnosis [22]. Besides, magnetic resonance imaging (MRI) is a highly sensitive approach, and immediate measures might be taken prior to the complication of the condition [23].

Despite the higher number of the evaluated cases in our series than the previously reported publications, one important limitation of our study is that we did not categorize our patients according to the size and location of the tear, and the success of the management protocol would be more likely to be depending on the size and approachability of the defect. Also, during 10 years, the operating surgeons, their experience, and the tissue adhesive alternatives have been changed and evolved. Therefore, it is not possible to provide a homogenous series during a decade of follow-up. However, we present the report of a single-center experienced on spinal procedures, and all cases underwent a similar procedure, single-level lumbar discectomy for the first time, and our data did not contain revision surgery cases.

## Conclusions

In conclusion, our findings showed that the presence of an incidental DT in patients who underwent a single level-level lumbar discectomy for the first time did not adversely affect clinical outcomes during 12 months of follow-up. Patients with DT were hospitalized for longer periods than compared to those without DT. Our findings suggest that the primary closure with suturing is superior to the use of hemostatic material and fibrin glue for the management of the dural defect.
